# Who Uses Mobile Phone Health Apps and Does Use Matter? A Secondary Data Analytics Approach

**DOI:** 10.2196/jmir.5604

**Published:** 2017-04-19

**Authors:** Jennifer K Carroll, Anne Moorhead, Raymond Bond, William G LeBlanc, Robert J Petrella, Kevin Fiscella

**Affiliations:** ^1^ Department of Family Medicine University of Colorado Aurora, CO United States; ^2^ School of Communication Ulster University Newtownabbey United Kingdom; ^3^ School of Computing & Maths University of Ulster Newtownabbey United Kingdom; ^4^ Lawson Health Research Institute Family Medicine, Kinesiology and Cardiology Western University London, ON Canada; ^5^ Family Medicine, Public Health Sciences and Community Health University of Rochester Medical Center Rochester, NY United States

**Keywords:** smartphone, cell phone, Internet, mobile applications, health promotion, health behavior

## Abstract

**Background:**

Mobile phone use and the adoption of healthy lifestyle software apps (“health apps”) are rapidly proliferating. There is limited information on the users of health apps in terms of their social demographic and health characteristics, intentions to change, and actual health behaviors.

**Objective:**

The objectives of our study were to (1) to describe the sociodemographic characteristics associated with health app use in a recent US nationally representative sample; (2) to assess the attitudinal and behavioral predictors of the use of health apps for health promotion; and (3) to examine the association between the use of health-related apps and meeting the recommended guidelines for fruit and vegetable intake and physical activity.

**Methods:**

Data on users of mobile devices and health apps were analyzed from the National Cancer Institute’s 2015 Health Information National Trends Survey (HINTS), which was designed to provide nationally representative estimates for health information in the United States and is publicly available on the Internet. We used multivariable logistic regression models to assess sociodemographic predictors of mobile device and health app use and examine the associations between app use, intentions to change behavior, and actual behavioral change for fruit and vegetable consumption, physical activity, and weight loss.

**Results:**

From the 3677 total HINTS respondents, older individuals (45-64 years, odds ratio, OR 0.56, 95% CI 0.47-68; 65+ years, OR 0.19, 95% CI 0.14-0.24), males (OR 0.80, 95% CI 0.66-0.94), and having degree (OR 2.83, 95% CI 2.18-3.70) or less than high school education (OR 0.43, 95% CI 0.24-0.72) were all significantly associated with a reduced likelihood of having adopted health apps. Similarly, both age and education were significant variables for predicting whether a person had adopted a mobile device, especially if that person was a college graduate (OR 3.30). Individuals with apps were significantly more likely to report intentions to improve fruit (63.8% with apps vs 58.5% without apps, P=.01) and vegetable (74.9% vs 64.3%, P<.01) consumption, physical activity (83.0% vs 65.4%, P<.01), and weight loss (83.4% vs 71.8%, P<.01). Individuals with apps were also more likely to meet recommendations for physical activity compared with those without a device or health apps (56.2% with apps vs 47.8% without apps, P<.01).

**Conclusions:**

The main users of health apps were individuals who were younger, had more education, reported excellent health, and had a higher income. Although differences persist for gender, age, and educational attainment, many individual sociodemographic factors are becoming less potent in influencing engagement with mobile devices and health app use. App use was associated with intentions to change diet and physical activity and meeting physical activity recommendations.

## Introduction

As of 2015, nearly two-thirds (64%) of the American public owned a mobile phone, which is an increase from 35% in 2011 [1]. It is estimated that 90% of the worldwide population will own a mobile phone by 2020 [1]. Current UK data reveals that mobile phone usage is increasing as 66% adults aged more than 18 years owned a mobile phone in 2015, up from 61% in 2014 [2]. Mobile phone ownership is higher among younger people, with 77% ownership for those aged 16-24 years [3]. Although mobile phone ownership is especially high among younger persons and those with higher educational attainment and income [4], those with lower income and educational attainment are now likely to be “mobile phone dependent,” meaning that they do not have broadband access at home and have few other options for Web-based access other than via mobile phone.

As mobile phone ownership rapidly proliferates, so does the number of mobile phone software apps grown in the marketplace [5]. Apps focused on health promotion are quite common: more than 100,000 health apps are available in the iTunes and Google Play stores [6]. This staggering number speaks to both the huge market and ongoing demand for new tools to help the public manage their diet, fitness, and weight-related goals, and the limitations of the current health care system to provide such resources. A recent study found that 53% of cell phone users owned a smartphone—this translates to 45% of all American adults—and that half of those (or about 1 in 4 Americans) have used their phone to look up health information [7]. There is increasing usage of health apps among health care professionals, patients and general public [8], and apps can play a role in patient education, disease self-management, remote monitoring of patients, and collection of dietary data [9-12]. Using mobile phones and apps, social media also can be easily accessed, and increasing numbers of individuals are using social media for health information with reported benefits and limitations [8].

Despite the massive uptake in mobile phone ownership and health app usage and their potential for improving health, important limitations of health apps are the lack of evidence of clinical effectiveness, lack of integration with the health care delivery system, the need for formal evaluation and review, and potential threats to safety and privacy [6,13-17]. Although previous studies have described the sociodemographic factors associated with mobile health and app use [7,18,19], it is a rapidly changing field with the most recent published reports reflecting data at least four to five years old. Additionally, there is a lack of information on the users of health apps in terms of their sociodemographic and health characteristics and health behaviors. Furthermore, to our knowledge, there have been no previous publications reporting on the association between the use of health apps, behavioral or attitudinal factors (ie, readiness or intentions to change), and health outcomes. This information is important for future health-improving initiatives and for identifying appropriate use of health apps among population groups.

Therefore, the aim for our study was 3-fold: (1) to describe the sociodemographic characteristics associated with health app use in a recent US nationally representative sample; (2) to assess the attitudinal and behavioral predictors of the use of health apps for health promotion; and (3) to examine the association between the use of health-related apps and meeting the recommended guidelines for fruit and vegetable intake and physical activity. Given the increasing focus on new models for integrating technology into health care and the need to expand the evidence base on the role of health apps for health and wellness promotion, these research questions are timely and relevant to inform the development of health app interventions.

## Methods

### Data Source

The National Cancer Institute’s Health Information National Trends Survey (HINTS) is a national probability sample of US adults that assesses usage and trends in health information access and understanding. HINTS was first administered in 2002-2003 as a cross-sectional survey of US civilians and noninstitutionalized adults. It has since been iteratively administered in 2003, 2005, 2008, 2011, 2012, 2013, and 2014. We used data from HINTS 4 Cycle 4 data released in June 2015, which corresponded to surveys administered in August-November, 2014. Publicly available datasets and information about methodology are available at the HINTS website [20]. The 2014 iteration reported herein contained questions about whether participants used mobile phone or tablet technology and software apps for health-related reasons. The overall response rate was 34.44%. This study was reviewed and qualified for an Exemption by the American Academy of Family Physicians Institutional Review Board.

### Participants

A total of 3677 individuals completed the 2014 HINTS survey. From this sample, 148 respondents were considered partial completers, in that they completed 50%-79% of the questions in Sections A and B. We included all 3677 respondents in our analysis. We used sampling weights from the HINTS dataset that were incorporated into the regression analyses.

### Measures

#### Demographics

We used participants’ self-report of their age, sex, race, ethnicity, income, level of education, English proficiency, height, and weight. We converted height and weight into body mass index (BMI), using weight (kg)/height (m^2^)×10,000, and classified participants as obese (≥30), overweight (29.9-26), or normal weight or underweight (<26).

#### Usage of Mobile Devices and Health Apps

We used participants’ responses to the 3 questions to characterize the distribution of subjects who used health-related software apps on their mobile devices. The participants were asked whether they had a tablet computer, smartphone, basic cell phone only, or none of the above. We examined factors for those with and without mobile devices, since previous studies have shown differences in seeking health information on the Internet related to access (eg, availability of a computer) [21,22], HINTS dataset is a nationally representative sample, and we wished to put our findings on app use in the larger population context. We categorized participants who had a mobile phone or a tablet device under the label “Device+.” Similarly, participants who did not report having a mobile phone or a tablet device were labeled “Device-.” Of the Device+ group, we also categorized them according to whether they had health apps on their device (Device+/App+) or did not have health apps on their device (Device+/App-).

#### Fruit and Vegetable Intake

We assessed fruit and vegetable intake using the 2 questions: amount of fruit consumed per day and amount of vegetables consumed per day (7 response options for each ranging from none to >4 cups per day). We reclassified the response options for both questions into a single dichotomous outcome variable, that is, the subject either (1) meets recommendations for fruit or vegetables (4 or more cups for each) or (2) does not meet recommendations for fruit or vegetables (all other response options). Fruit and vegetable scores were analyzed separately.

#### Physical Activity

We assessed physical activity using the 2 questions: (1) in a typical week how many days do you do any physical activity or exercise of at least moderate intensity, such as brisk walking, bicycling at a regular pace, and swimming at a regular pace? (8 response options ranging from none to 7 days per week) and (2) on the days that you do any physical activity or exercise of at least moderate intensity how long do you do these activities? (2 response options for minutes and hours). We reclassified the response options into a single dichotomous outcome variable for physical activity, that is, whether the subject (1) met physical activity recommendations (≥150 minutes per week) or did not meet the physical activity recommendations (<150 minutes per week).

#### Intentions to Change Behavior

We examined participants’ intentions to change behavior based on the 5 questions (all with yes or no responses): At any time in the last year, have you intentionally tried to (1) increase the amount of fruit or 100% fruit juice you eat or drink, (2) increase the amount of vegetables or 100% vegetable juice you eat or drink, (3) decrease the amount of regular soda or pop you usually drink in a week, (4) lose weight, and (5) increase the amount of exercise you get in a typical week?

### Statistical Analysis

The outcome variable (OUTCOME) was a composite derived from 3 survey variables: (1) *own a smartphone (an Internet-enabled mobile phone “such as iPhone android BlackBerry or Windows phone” differentiated from a “basic cell phone,” hereafter referred to as “mobile phone”) or device*, (2) *have health apps on mobile phone or device*, and (3) *use of health apps*. *Own a mobile phone or device* was a system-supplied derived variable to categorize responses given to question B4 (possession of a mobile phone or tablet device). *Have health apps on mobile phone or device* (question B5) asked about health apps on a tablet or mobile phone. *Use of health apps* (question B6a) asked whether the apps on a mobile phone or tablet helped in achieving a health-related goal. OUTCOME consisted of 3 levels: Device-/App- (33.2% of respondents), Device+/App- (44% of respondents), and Device+/App+ (22.77% of respondents). Device referred to having a tablet or mobile phone, and App referred to having a health-related app that ran on a tablet or mobile phone. A total of 93 of 3677 respondents were unable to be classified due to missing data. These people were not used in the analyses. To assess the relationship between OUTCOME and the demographic or health behavior variables, simple unweighted 2-way crosstab tables were generated and tested with a chi-square test of association. We used a cutoff of *P*<.05 to determine statistical significance for all analyses.

We used the R programming language (R-Studio) and SPSS (SPSS Inc) for all data modeling and analysis carried out in this study.

## Results

### Principal Findings

**Figure 1:**
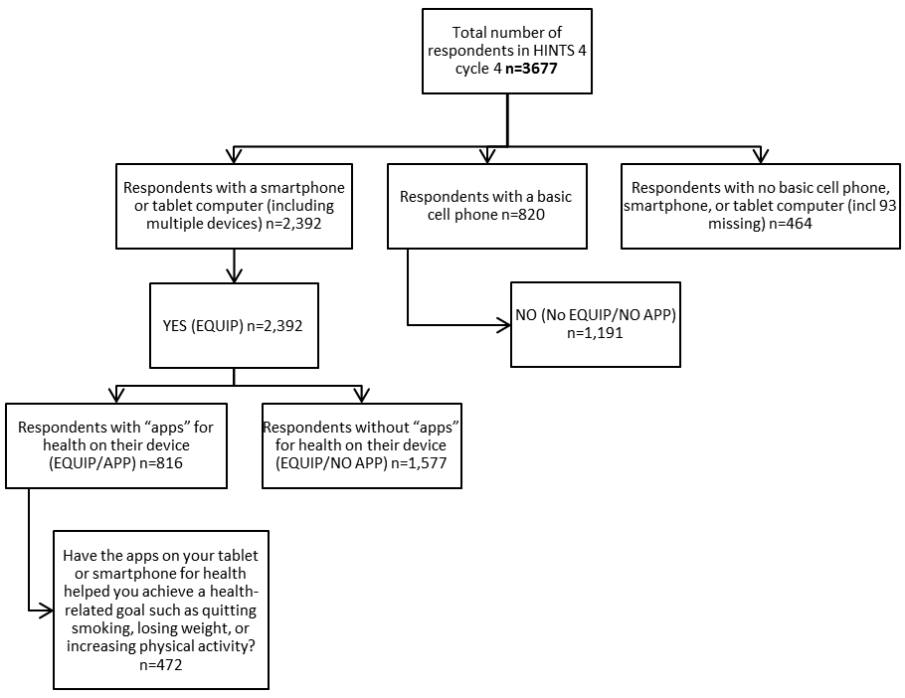
Health Information National Trends Survey (HINTS) respondents’ use of mobile phones, tablets, and apps.

From the 3677 total HINTS respondents, 3584 answered questions about whether or not they had a tablet computer or mobile phone, or used apps. Figure 1 shows the participants in this study.

### Demographic Variables Associated With App Use

Table 1 compares respondents grouped into Device+/App+, Device+/App-, and Device-, according to sociodemographic characteristics. As shown in Table 1, those who used health apps (compared with those who either did not have apps or did not have the necessary equipment) were more likely to be younger, live in metropolitan areas, have more education, have higher income, speak English well, be Asian, and report excellent health. There was no significant association between both BMI and smoking status and app use.

**Table 1:** Demographic variables associated with app usage.

Demographic variables	Device+/App+ n^b,c^ (%)^d^	Device+/App- n (%)	Device- n (%)	*P* value
Sex (female vs male; n^a,c^=3519)	808 (51.62)	1555 (50.23)	1156 (55.29)	.39
Age (18-44 years vs 45+ years; n=3415)	782 (65.62)	1552 (52.25)	1111 (21.92)	<.001
Education (high school or less vs some college or college graduate, n=3444)	788 (12.72)	1535 (27.95)	1121 (51.82)	<.01
Income (US $0-49,999 vs 50,000 or greater; n=3530)	808 (31.72)	1560 (42.20)	1162 (75.12)	<.001
Race or ethnicity (white vs other; n=3273)	763 (71.85)	1453 (78.52)	1057 (83.68)	<.01
BMI (normal vs overweight, obese; n=3420)	782 (33.71)	1524 (36.98)	1114 (33.82)	.49
Metro vs nonmetro (n=3584)	816 (92.10)	1577 (85.67)	1191 (78.93)	<.001
Speak English (very well or well vs not well or not at all; n=3584)	759 (99.37)	1497 (97.13)	1089 (90.37)	<.001
Self-rated health (excellent, very good, good vs fair or poor; n=3477)	795 (92.85)	1544 (89.74)	1138 (74.99)	<.001

^a^The sample sizes (n’s) listed for each variable in the far left column represent the total number of respondents across all app-usage categories (Device+/App+, Device +/App-, Device-) who answered that question.

^b^The sample sizes (n’s) listed for each variable within each cell represent the total number of respondents within a given app-usage category (either Device+/App+, Device +/App-, or Device-) who answered that question.

^c^Sample sizes vary for each variable due to missing values.

^d^Population estimates were used for the numerators and denominators in the calculation of percentages. Row percentages do not add to 100%, as the table shows percentages within a given app-usage category (Device+/App+, Device +/App-, or Device-).

### Association Between the Use of Apps and Intentions to Change Diet, Perform Physical Activity, and Lose Weight

Table 2 shows the association between the use of apps (versus Device+/App- or Device-) with intentions to change diet, perform physical activity, or lose weight. As Table 2 shows, participants with apps were significantly more likely to report intentions to improve fruit (*P*=.01) and vegetable consumption (*P*<.01), physical activity (*P*<.01), and weight loss (*P*<.01) compared with those in the Device+/App- or Device- groups.

**Table 2:** Association between the usage of apps for health-related goal and intentions to change diet, physical activity, or lose weight.

Health-related intention	Device+/App+ n (%)	Device+/App- n (%)	Device- n (%)	*P* value^a^
Increase fruit	545 (63.76)	885 (58.50)	654 (48.94)	.01
Increase vegetables	621 (74.92)	1023 (64.26)	717 (50.02)	<.01
Decrease soda	630 (84.96)	1135 (82.76)	754 (77.36)	.06
Increase physical activity	707 (82.99)	1237 (65.42)	769 (49.94)	<.01
Lose weight	692 (83.36)	1259 (71.75)	881 (60.02)	<.01

^a^Significance between participants with apps (Device+/App+) compared with those not using apps or devices (Device+/App- or Device- groups).

### Association Between the Use of Apps and Meeting Recommendations for Fruit and Vegetable Intake and Physical Activity

Table 3 shows the association between the use of apps (versus Device+/App- or Device-) and meeting the recommendations for fruit and vegetable intake and physical activity. Participants in the Device+/App+ group were not significantly more likely to meet recommendations for fruit and vegetables compared with those in the Device+/App- or Device- groups; however, they were significantly more likely to exercise more than 2 hours per week.

**Table 3:** Association between the use of apps for health-related goal and meeting recommendations for fruit and vegetables and physical activity.

Percent respondents meeting recommendations	Device+/App+ n (%)	Device+/App- n (%)	Device- n (%)	*P* value^a^
Fruit	804 (8.87)	1560 (7.96)	1161 (5.43)	.25
Vegetables	809 (4.81)	1557 (3.01)	1155 (3.48)	.27
Physical activity	801 (56.23)	1552 (47.79)	1144 (37.69)	<.01

^a^Significance between participants with apps (Device+/App+) compared with those not using apps or devices (Device+/App- or Device- groups).

### Predicting Health App Adoption Only (Binary Classification)

Table 4 presents the statistically significant odds ratios (ORs) as derived using multivariate logistic regression when applied to the entire dataset. As expected, those aged 45-64 years (OR 0.56) or 65+ years (OR 0.19) had a reduced likelihood of having adopted health apps relative to younger persons. It also showed that males were slightly less likely (OR 0.80) to have a health app compared with females. The most significant finding was the confirmation that graduates had significantly higher odds (OR 2.83) of having a health app especially when compared with those who had attained an education that was considered “less than high school” (OR 0.43). The results also indicated that the category “completed high school only” had no predictive ability for estimating whether a person had adopted a health app.

**Table 4:** Statistically significant odds ratios derived using multivariate logistic regression when applied to the entire dataset for predicting health app adoption only.

Variable	Odds ratio (95% CI)	*P* value
Age (45-64 years)	0.56 (0.47-0.68)	<.001
Age (65+ years)	0.19 (0.14-0.24)	<.001
Sex (male)	0.80 (0.66-0.94)	<.01
Education (college graduate or higher)	2.83 (2.18-3.70)	<.001
Education (less than high school)	0.43 (0.24-0.72)	<.01
Education (some college)	1.70 (1.30-2.26)	<.01
Race (black)	1.25 (0.99-1.55)	.05

### Predicting Mobile Technology Adoption Only (Binary Classification)

Table 5 presents the statistically significant ORs that increased or decreased the likelihood that a person had adopted mobile technology (tablet or mobile phone). Interestingly, there were no statistically significant ORs for gender or racial categories. However, similar to predicting health app adoption, both age and education were significant variables for predicting whether a person had adopted a mobile device, especially if that person was a college graduate (OR 3.30). In addition, the results indicated that the category “completed high school only” had no predictive ability for estimating whether a person had adopted a mobile device.

**Table 5:** Statistically significant odds ratios derived using multivariate logistic regression when applied to the entire dataset for predicting mobile device adoption only.

Variable	Odds ratio (95% CI)	*P* value
Age (45-64 years)	0.35 (0.28-0.45)	<.001
Age (65+ years)	0.09 (0.07-0.12)	<.001
Education (college graduate or higher)	3.30 (2.65-4.11)	<.001
Education (less than high school)	0.51 (0.37-0.70)	<.001
Education (some college)	1.87 (1.50-2.32)	<.001

## Discussion

### Principal Findings

Our first objective was to describe the sociodemographic and health behavior characteristics associated with health app use in a recent US nationally representative sample. Consistent with previous findings [7], we found that those who were younger, had more education, reported excellent health, and had a higher income were more likely to use health apps. Our predictive modeling using multivariate logistic regression showed that education, sex, gender, and race were only mildly to moderately potent in predicting mobile technology adoption.

Our second objective was to assess the behavioral and attitudinal predictors of the use of health apps for health promotion. We found that participants with apps were also more likely to report intentions to improve fruit and vegetable consumption, physical activity, and weight loss. Finally, the third objective was to examine the association between the use of health-related apps and meeting the recommended guidelines for fruit and vegetable intake and physical activity. We found that participants in the health apps group were significantly more likely to meet recommendations for physical activity compared with those without a device or health apps.

### Comparison With Prior Work

This study shares some similarities with previous HINTS analyses. For example, McCully et al [19] reported that users of the Internet for diet, weight, and physical activity tended to be younger and more educated and that Internet use for these purposes was more likely to be associated with higher fruit and vegetable intake and moderate exercise. However in that study, women were no more likely than men to use the Internet for diet, weight, and physical activity, which was different from our findings. In that study, minorities were more likely to use the Internet; in our study, we found no such association. Consistent with our findings, Kontos et al found that males, those with lower education, and older US adults were less likely to engage in a number of eHealth activities [18]. Similar to their findings 3 years ago, our findings pointed to differences by education for app use for health promotion.

The association between app use, intention to change lifestyle behaviors, and actually meeting recommendations for healthy lifestyle factors is interesting and could be due to several reasons. First, it is possible that there are preexisting differences in individuals who engage with health apps compared with those who do not. Users of health apps may have greater motivation and interest in changing their diet, weight, or physical activity. A recent review found that very few available apps provided evidence-based support to meet lifestyle recommendations [13]. It could also be that app users are engaging with health apps to help them simply track or self-manage differently than their counterparts; thus, there could be differences in preferences or needs. Due to the correlational nature of the data, we cannot draw conclusions about the relationships or causal pathways. Similar observations have been reported in a study of users of the Internet for diet, weight, and physical activity promotion [19].

The prevalence of app usage in our study was 22% (816/3677). This is a doubling from the Kontos study in which 11.7% downloaded info onto a mobile device. Although the questions in these 2 HINTS datasets were worded differently (eg, “downloaded” is broader and not referring exclusively to downloading an app), it suggests that demand for apps continues to rise and offers potential for reaching a growing segment of the US population.

Our findings provide evidence for educational, age, and gender differences in the use of mobile devices and health apps. Educational attainment, age, and gender have been previously shown to be important predictors of adoption of mobile devices and apps [18]. Educational attainment appears more important than other variables commonly used as proxies for socioeconomic position (eg, income, race or ethnicity). The reasons for the educational differences are unclear, but may reflect skills and confidence with the use of devices and possibly social norms related to perceived value. Similarly, age likely reflects both social norms and cohort effects, that is, exposure during younger ages to these devices and apps. The reasons for gender differences are less clear, but may reflect differences in health-seeking behavior, and interest and participation in healthy lifestyle interventions generally.

### Limitations

This study had limitations that should be kept in mind when interpreting results. First, HINTS is a cross-sectional survey; although it is a nationally representative cohort of individuals, we were not able to evaluate the trends in an individual’s health app use over time. There is the possibility of unmeasured confounding, that is, unidentified factors that might be associated with app use and intentions or health behaviors, which could influence the interpretation of results. Although the results showed association, it did not indicate a causal relationship. This study could not answer the question of whether more motivated individuals sought out apps, or whether app use improved motivation and health outcomes. Furthermore, some of the cells for subgroups were small, thereby limiting the generalizability of some of the subanalyses. As with all cross-sectional surveys, this was a study of association, not causation. Finally, we were limited by the questions that were asked in the HINTS survey. For example, we did not have details about specific health apps or features of apps used, the intensity of use, whether the apps were interactive and linked to other health promotion supports (eg, telehealth), and other strategies used for health behavior change. Despite these limitations, the results did identify areas for future research and add to the knowledge base about predictors of the use of health apps.

### Conclusions

Compared with previous studies, many individual sociodemographic factors are becoming less important in influencing engagement with mobile devices and health app use; however, differences persist for gender, age, and educational attainment. As health care undergoes technological transformation with its electronic health records systems and individuals’ access to their records, there are many opportunities for clinical care models to be expanded and improved, perhaps through the use of apps as a means for sharing data, although this remains an unanswered question. This study contributes to the literature by providing up-to-date information on populations most and least likely to use health apps to guide clinical interventions, commercial developers, and public health programs when designing eHealth technology.

## Abbreviations

HINTS: Health Information National Trends Survey
